# Doxorubicin-loaded NK exosomes enable cytotoxicity against triple-negative breast cancer spheroids

**DOI:** 10.22038/ijbms.2024.79378.17194

**Published:** 2024

**Authors:** Zahra Sadat Hashemi, Mahlegha Ghavami, Fateme Mohammadi, Mahdieh Shokrollahi Barough, Farhad Shokati, Saber Asghari, Saeed Khalili, Mandana Akbari Yekta, Ardeshir Ghavamzadeh, Ramin Sarrami Forooshani

**Affiliations:** 1 ATMP Department, Breast Cancer Research Center, Motamed Cancer Institute, ACECR, Tehran, Iran; 2 Pathology Department, Dalhousie University, Halifax, Canada; 3 Department of Hematology, School of Allied Medical Sciences, Tehran University of Medical Sciences, Tehran, Iran; 4 Biomaterials and Tissue Engineering Department, Breast Cancer Research Center, Motamed Cancer Institute, ACECR, Tehran, Iran; 5 Department of Biology Sciences, Shahid Rajaee Teacher Training University, Tehran, Iran; 6 Cancer and Cell Therapy Research Center, Tehran University of Medical Sciences, Tehran, Iran

**Keywords:** Breast cancer, Cellular spheroid, Chemotherapy, Exosome, Natural killer cells

## Abstract

**Objective(s):**

Natural killer (NK) cells are the most professional innate immune cells that initiate extracellular apoptosis via cytotoxic granules in malignant cells. Antitumoral properties of NK-derived exosomes (Exos) are attributed to their parent cells. Loading drugs into Exos as a carrier can enhance their effect and enable targeted delivery. In the present study, we aim to deliver Doxorubicin (DOX) to the breast cancer spheroids by NK-Exos.

**Materials and Methods:**

Peripheral blood mononuclear cells (PBMC) were used to harvest NK cells, and NK-Exos were isolated from NK cell expansion medium using an Exo-spinTM kit. DOX was loaded via the ultrasonication method. AO/EtBr, Annexin/PI, DAPI, MTT, and spheroids of human breast cancer were used to track the cytotoxic effect of DOX-NK-Exos. The colony formation assay, scratch and transwell assays, Real-Time PCR for p53 and VEGF-A, and WB for protein expression were also performed.

**Results:**

When compared to free DOX, all viability tests validated the inhibitory effects of DOX-NK-Exos. The obtained results indicated that DOX-NK-Exos selectively reduced tumor cell viability and spared fibroblast and MCF-10A as noncancerous cells. Long after spheroid treatment, DOX-NK-Exos’ remarkable effect persisted.

**Conclusion:**

Human breast carcinoma mass treated with DOX-NK-Exos underwent apoptosis and showed a strong inhibitory effect on proliferation. Thus, they can reduce the side effects of chemotherapeutics and can be used as drug carriers with selective toxicity. Additionally, the additive action of this combination formula results in a more severe loss in cell viability.

## Introduction

One of the most widely used therapeutic approaches for the treatment of cancer is still chemotherapy. Doxorubicin (DOX) is one of the highly potent and synthetic antineoplastic agents. This molecule exerts its effects by inhibiting topoisomerase II, which unwinds the DNA and relaxes the supercoils of DNA during transcription (1). This chemotherapeutic agent is commonly used to treat breast cancer and solid tumors. However, its clinical use is highly limited due to its dose-dependent toxicity and biocompatibility (2). One of the most dangerous types of breast cancer is still thought to be triple-negative breast cancer (TNBC), which has no specific treatments and a dismal prognosis (3). The only systemic treatment modalities for TNBC are platinum chemotherapy (4) and untargeted chemotherapy (single or combined). Since chemotherapy operates by targeting the rapid division of cancerous cells, it influences healthy normal cells with similar high proliferation rates, including those found in bone marrow, hair follicles, and the gastrointestinal tract. This dual effect leads to the typical secondary severe side effects associated with chemotherapy (5, 6). Thus, these untargeted strategies offer limited options, and given these circumstances, a safe and efficient targeted delivery platform based on the drug delivery system (DDS) for TNBC therapy is required (7). To have an effective passive and active targeted cancer therapy, many cytotoxic drug carriers have been proposed, including carbon nanotubes, polymeric micelles, liposomes, dendrimers, polymeric conjugates, and polymeric nanoparticles, to improve the permeability and retention of the drug.

Exosomes (Exos) are natural membrane-bound extracellular vesicles (EVs) and are generally smaller than other EVs, from about 50 to 150 nanometres (nm) in diameter (8). Hence, Exos could be considered nanovesicles that offer distinct advantages, such as low immunogenicity and toxicity, high biocompatibility (9, 10), and the ability to cross the blood-brain barrier (2, 11). These nanovesicles are suggested as efficient alternatives to synthetic drug carriers. Several studies have exploited exosomes to deliver chemotherapeutics in breast cancer (e.g., paclitaxel (PTX) and DOX), nucleic acids (e.g., siRNA and miRNA), and proteins (12-14). These nanovesicles could be the derived exosomes by naturally biological biogenesis or the modified exosomes by different strategies such as pH gradient/surface charge, ligand-receptor binding, or magnetism-guided (15, 16).

Exos-mediated encapsulation of DOX could significantly reduce the adverse side effects on other organs and specifically deliver the drug to the target tissue (9). Several studies have already addressed the DOX-loaded Exos. Mesenchymal stem cells (MSCs) were the main parental cells employed for exosome generation. Gomari *et al*. have employed MSC-Exos to encapsulate DOX and reduce the tumor growth rate of HER2-positive breast cancer (17). Interestingly, overexpression of human epidermal growth factor receptor 2 (HER2) has been found in up to 30% of breast cancer tumors (18).

Despite the numerous advantages of two-dimensional (2D) cell cultures, such as ease of use and cost-effectiveness, there are also some limitations, such as reduced cell-cell communication (19). Three-dimensional (3D) scaffolds play a pivotal role in the examination of drug loading and controlled delivery of Exos (20, 21). There are no physical limitations to the delivery of nanoparticles in a monolayer cell culture. In contrast, drug diffusion profiles are essentially altered by the three-dimensional organization of a tumor mass, thanks to interactions between cells and matrices (22). Several studies have developed spheroids or organoids from tumor cells to explore the therapeutic effects of exosomes. Spheroids are the most common models to test drug-loaded EVs (23). In the present study, we aimed to evaluate our treatment strategy in 3D cultures. The arrangement of cells in 3D conformations would provide a better physiological model for drug treatment and preserve physiological cancer characteristics (24).

Exos specifically package cytoplasmic and membranous contents of their parent cells during secretion. Therefore, the choice of exosome source is pivotal for clinical use. Natural killer cells (NK) are classified as innate lymphoid cells. They play a critical role in the immune responses against tumors and viral infections (25, 26). They efficiently treated hematologic cancers like acute myeloid leukemia and metastatic breast cancer (27, 28). Since NK cells participate in specific and nonspecific immunity, several studies have indicated the antitumor effects of Exos derived from NK cells (NK-Exos). NK cells have been demonstrated to exert their antitumor activity via EVs; therefore, they could provide desirable properties as a source of exosomes. Unlike other lymphocytes, NK cells constitutively secrete Exos, regardless of their resting or activated status (29). Zhu *et al*. have demonstrated that NK-Exos can have a potential therapeutic effect against aggressive melanoma cells (30). They have shown that NK-Exos could inhibit the abnormal proliferation of melanoma cells and increase the survival rate of mouse models of melanoma. NK-Exos were found to contain perforin and killer proteins (i.e., Fas ligand (FasL)) that inhibit cancer growth (31). Thus, NK-Exos could contain immunologically active components that exert cytotoxicity against tumor cells. It means that the natural inhibitory and toxic effects of NK cells accompany the loading capacity of NK-Exos.

In the present study, NK-Exos were used to load and deliver DOX molecules. The study aims to determine the antitumor efficacy of DOX-loaded NK-Exos (in a 3D model (spheroid) of breast cancer cells) compared to the free DOX.

**Figure 1:**
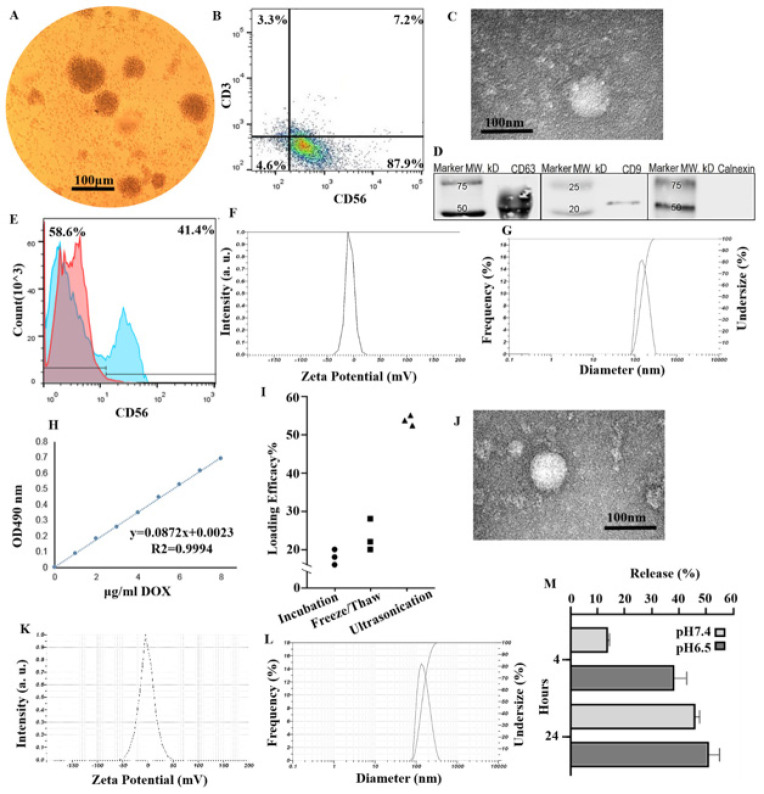
Exosome characterization

A) NK cell morphology in colony expansion, B) NK cell purity in case of CD3 and CD56 expression after 21 days in specific media. (C) TEM of NK-Exos, scale bar=100nm. D) The positive expression of exosome common markers CD9 and CD63 as the general Exos markers and non-expression of Calnexin as the endoplasmic reticulum marker in exosomes lysate analyzed using western blot. E) The expression of NK-Exos specific proteins (CD56) was analyzed using flow cytometry. F) The zeta potential was obtained by the zeta sizer nano ZS100 for NK-Exos. G) Particle size of NK-Exos was analyzed using DLS and the peak diameter was 131.2 nm, which corresponds to exosomes. H) Standard curve of DOX, I) the loading efficiency of DOX in exosomes by ultrasonication, freeze/thaw, and incubation. The ultrasonication showed the highest encapsulation efficiency. J) TEM image of DOX-NK-Exos, there were not seen any significant changes in the morphology of DOX-loaded Exos. Scale bar=100nm. K) Zeta potential of DOX-NK-Exos showed a small increase in the negative surface charge. L) The average diameter of DOX-NK-Exos was 140.5nm showing no significant aggregation or size increase after DOX loading. M) DOX was released from Exos and the release rate was determined by dialysis membrane. Data were presented as means±SD of three separate experiments, n=3 and *P<0.05.

NK: Natural killer cells; Exos: Exosomes; TEM: Transmission electron microscopy; DOX: Doxorubicin

**Figure 2:**
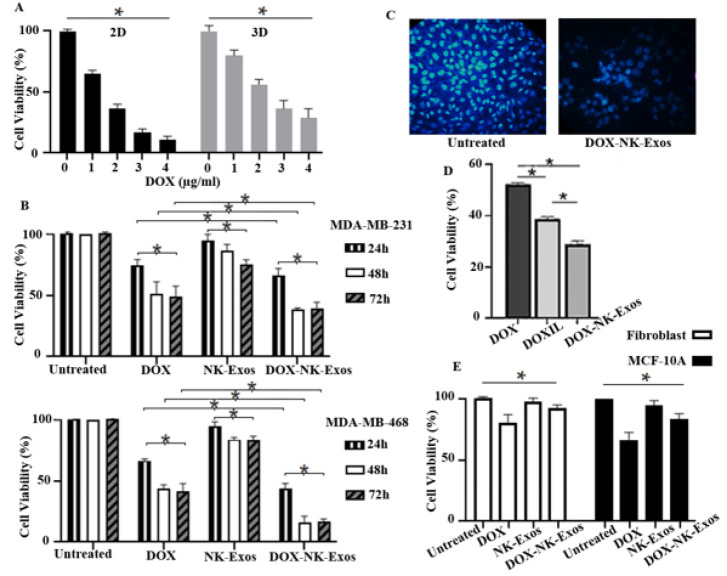
Cytotoxicity of DOX-NK-Exos compared to NK-Exos and free DOX

A) Calculation of IC_50_ for DOX using MTT-dose response in 2D and 3D culture of MDA-MB-231 were 1.8µg/ml and 2.2µg/ml, respectively. B) The results of the MTT assay for MDA-MB-231 and MDA-MB-468, indicated significant toxicity of DOX-NK-Exos after 24, 48, and 72 hr. C) Survival rate of MDA-MB-231 cells at 48 hr detected using DAPI staining. D) Simultaneous comparison of DOX (2.2 µg/ml), DOXIL (contained 2.2 µg/ml DOX as effective material, and DOX-NK-Exos (contained 2.2 µg/ml DOX) treatment on MDA-MB-231 spheroids. E) The effect of DOX-NK-Exos on fibroblast as primary cells and MCF-10A as the control cell line, after 48 hr, were visualized by MTT assay. DOX-NK-Exos showed preferential cytotoxicity against cancer cells and minimum cytotoxicity against noncancerous normal cell lines. Data were presented as means±SD of three separate experiments, n=3, *P<0.05. DOX: Doxorubicin; NK: Natural killer; Exos: Exosomes

**Figure 3:**
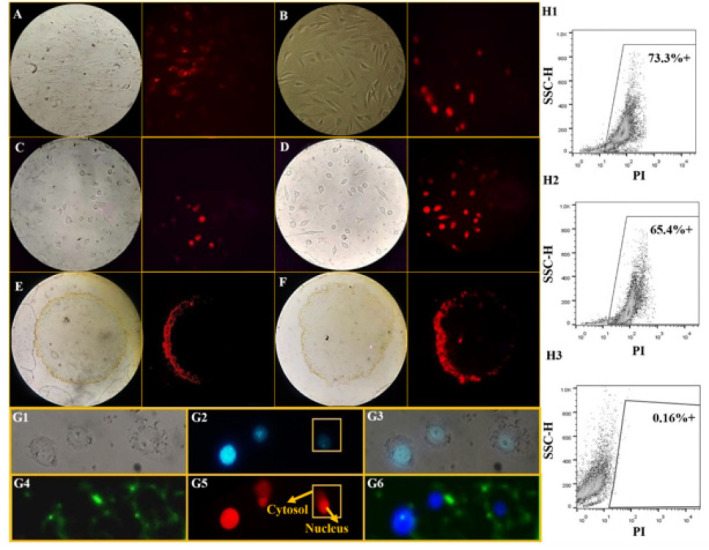
Binding and Uptake of DOX and DOX-NK-Exos by MDA-MB-231 cells and non-cancerous cell lines in 2D and 3D cultures

Uptake of DOX-NK-Exos (red) by fibroblast (A) and MCF-10A (B). Uptake of DOX (red) (C and E) and DOX-NK-Exos (D and F) by MDA-MB-231 cells in 2D and 3D cultures, respectively. DOX-NK-Exos can be more effectively taken up by the cells in both 2D and 3D cultures (magnification 40X): G1) Bright-field microscopy of MDA-MB-231 cells treated by DOX-NK-Exos, G2) dyed nuclei of cells by DAPI staining (blue). G3) merged the picture of G1 and G2. G4) Binding of PKH-67 labeled DOX-NK-Exos (green) to cells. G5) DOX (originated from DOX-NK-Exos) entered the cells and it was completely clear that DOX accumulated in the nucleus. G6) merged the picture of G4 and G2. Uptake of DOX-NK-Exos (H1) and DOX (H2) compared to the NK-Exos group (H3) were quantified using flow cytometry analysis in the PI channel. DOX-NK-Exos showed higher binding and uptake to MDA-MB-231 cells compared with NK-Exos. The cellular uptake of DOX-NK-Exos has a shift in mean peak fluorescence intensity signals as compared with NK-Exos. The percentages indicated the proportion of MDA-MB-231 cells that have interacted with DOX-NK-Exos.

NK: Natural killer cells; Exos: Exosomes; DOX: Doxorubicin

**Figure 4:**
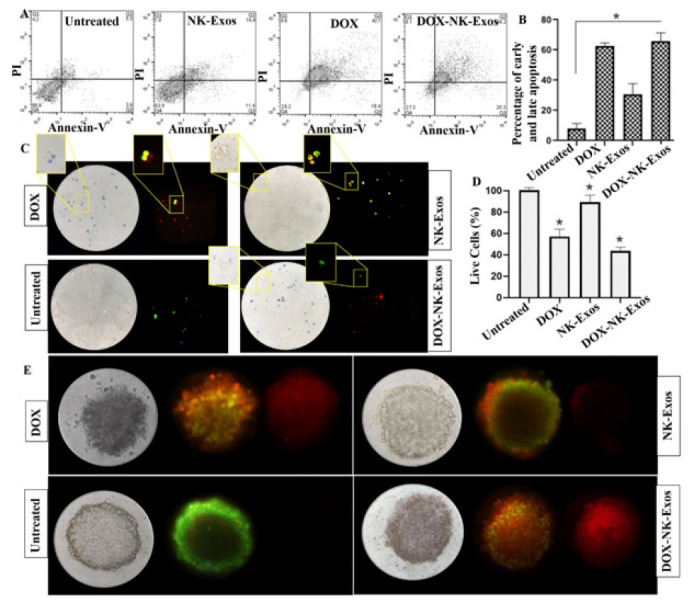
The effect of DOX-loaded exosomes on the cell apoptosis

A) Apoptosis rate of MDA-MB-231 cells using flow cytometry (Q1: Necrosis, Q2: Late Apoptosis, Q3: Early Apoptosis, Q4: Live Cells). B) Data (Late and Early Apoptosis) were presented as means ±SD of three separate experiments, n=3, *P<0.05 vs untreated group. C) The cells in 2D were stained with AO and EtBr, and images were captured under the fluorescent microscope. Green, red, and orange-red fluorescence indicated live, dead, and apoptosis cells, respectively. Also, the cells were stained with trypan blue as a viability color under the bright-field microscopy (blue as dead cells). The D graph showed quantified values. Data were presented as means ±SD of three separate experiments, n=3, *P<0.05 vs untreated groups. E) AO and EtBr staining of spheroids. The data shown were from a representative experiment. AO: Acridine orange, EtBr: Ethidium bromide; DOX: Doxorubicin

**Figure 5:**
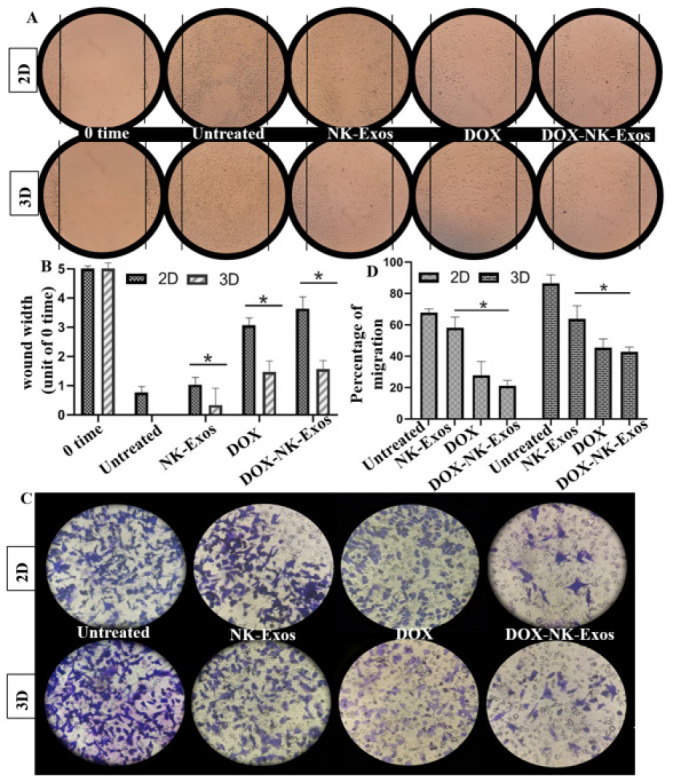
The Effect of DOX-loaded exosomes on cell mobility and migration

A) Wound Healing Assay: movement of MDA-MB-231 cells derived 2D and 3D cultures into the wound was shown for 0time and after 24h of scratching in all groups. B) Quantification of the wound healing assay based on the wound width (the distance between two edges) at 0time. The wound healing was slower in the DOX-NK-Exos treated. C) The migration abilities of cells for 2D and 3D were assessed using the Transwell assay. Subsequently, migrated cells in untreated, NK-Exos, DOX, and DOX-NK-Exos treated were stained with crystal violet solution and were counted using ImageJ software. DOX-NK-Exos suppressed the migration of MDA-MB-231 in the transwell assay higher than in other treatment groups. The D graph shows quantified values. Data shown were from a representative experiment and were presented as means ±SD of three separate experiments, n=3, *P<0.05 vs untreated groups.

DOX: Doxorubicin; NK: Natural killer; Exos: Exosomes

**Figure 6:**
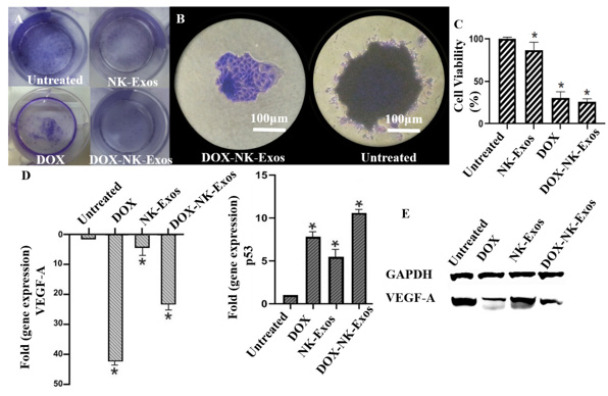
The effect of DOX-NK-Exos in long term and gene expression

A) The suspended single cells derived untreated, NK-Exos, DOX, and DOX-NK-Exos treated spheroids were incubated for one week. After fixation, the former colonies were stained with crystal violet. B) A single colony originated from a single cell in DOX-NK-Exos treated and untreated groups. The C graph is the quantification results using the absorbance of colored colonies and is relative to the untreated MDA-MB-231 (maximum absorbance) as 100. Data were presented as means ±SD of three separate experiments, n=3, *P<0.05 vs untreated MDA-MB-231. D) The expression levels of p53 and VEGF-A genes were examined in treated groups compared to the untreated groups by Real-Time PCR (Pfaffl ratio) and normalized to β-actin expression level. VEGF-A gene expression was downregulated in MDA-MB-231 cells that were treated with DOX, DOX-NK-Exos but reversely p53 gene expression was upregulated in treated groups. Data were presented as means ±SD of three separate experiments, n=3, *P<0.05 vs untreated groups. E) The protein level of VEGF-A in treated groups compared to the untreated group by western blot (normalized to GAPDH).

DOX: Doxorubicin; NK: Natural killer; Exos: Exosomes

## Materials and Methods

***NK cell isolation and expansion***

Using Ficoll solution (GE Health Care-Sweden) and a high-density-based centrifuge, whole peripheral blood mononuclear cells (PBMCs) of a healthy participant were isolated. The details of the project were explained to the participants. Based on the requirements assigned by the Medical Ethics Committee (IR.ACECR.JDM.REC.1400.054), each participant signed a written consent form before recruitment. According to the instruction of the CD56 negative selection kit, CD56 negative pooled biotin-labeled antibodies and total PBMCs were mixed together. The mixture was then exposed to the streptavidin-labeled magnetic beads. Passing through the supra-magnetic column of MACS (Milteny Biotech, USA) was the next step for the cell suspension. CD56 positive NK cells were suspended gently into 1000 IU/ml recombinant human IL-2 (R&D system-USA), 10 ng/ml of NK cell activation supplement containing MACSiBead™ Particles (Milteny biotech-USA), and RPMI1640 culture medium (Gibco-USA) containing 5% human AB serum (Sigma-Germany). Finally, the total isolated cells were seeded in a T25 non-treated flask. Refreshment of the conditioned media, containing 500 IU/ml rhIL-2, was continued up to 21 days every three days (32). Additionally, the cells were treated with PE-labeled anti-CD56 (Biolegend-USA) and FITC-labeled anti-CD3 on the twenty-first day, after which the Attune NxT flow cytometry instrument (with FlowjoTM 10.8 software) was used to read the results.

***Isolation and characterization of NK-Exos***

The donor serum was placed in an Amicon filter (100 kDa, Merck Millipore) and centrifuged at 4,000 g for 10 min. On the 21st day, pure NK culture was conducted using the passed-through fraction, which included the Exos-free serum. Following a two-day period, the NK cell’s medium was extracted, and the resultant Exos were purified in accordance with the manufacturer’s instructions using the Exo-spinTM kit (Cell Guidance Systems). Bradford protein assay (Fermentas) was used to quantify the concentration of exosomes (26). 

Zeta potential and exosome particle size were determined using a dynamic light scattering (DLS) system (HORIBA-SZ100 Instruments). Isolated NK-Exos were analyzed for their particle size using a transmission electron microscope (TEM) (PLILIPs, EM208S, The Netherlands). Moreover, following a separation step on a 12.5% SDS-PAGE, 10 μg of total NK-Exos was transferred to nitrocellulose membranes (PROTRAN, Schleicher & Schuell BioScience, Germany). Membranes were blocked with 3% skim milk. Then, the membranes were incubated with HRP-conjugated antibodies (anti-CD9, Calnexin, and CD63 at a dilution of 1:3000 overnight at 4 °C). After washing with TBST buffer, the membranes were ultimately analyzed using the DAB substrate. Finally, flow cytometry was employed to identify the CD56-positive Exos using a biotinylated anti-CD56 antibody (eBiosciences-USA). 

***Loading and release DOX***

The different concentrations (0–8 µg/ml) of DOX (Doxorubicin Hydrochloride or DOX.HCl, 2 mg/ml) up to 100 µl volume PBS were added to 96-well plates. An enzyme-linked immunosorbent assay (ELISA) plate reader measured the optical density (OD) based on the intrinsic fluorescence of DOX (standard curve). The OD measurement was done at a wavelength of 490 nm. NK-Exos and DOX were mixed in a 1:1 (100: 100 μg) ratio and loaded by different methods: ultrasonication (QSonica Q700 with MicroTip, 20%amplitude, six cycles of the 30 sec on/off with 2 min cooling period between each cycle), incubation (2 hr at 22 °C by shaking), and freeze/thawing. The mixture was then eluted through an Amicon filter (100 kDa) to remove the excess unloaded DOX, which was quantified by calculating the UV-Vis absorbance based on the standard curve (33):

DOX loading efficiency [%] =100–(W. recovered DOX in eluate/W. total DOX×100)

By transferring the DOX-NK-Exos into a dialysis membrane (Sigma, 14 kDa MWCO), the stability and release profile of DOX were investigated. The DOX-NK-Exos were added to 100 ml of 10 mM PBS (pH 7.4 and pH 6.5) and stirred at room temperature. The released DOX out of the dialysis membrane was quantified based on the standard curve.

As an alternative, 50% FBS (Hyclone Laboratories, Logan, UT, USA) was incubated at 37 °C for 24 hr with DOX-NK-Exos, and the mixture was eluted using an Amicon filter (100 kDa). The DOX recovered in the eluate was also quantified.

DOX instability or release [%] =W. the DOX in the medium outside dialysis or the recovered eluate/W. the total DOX in post-incubation×100.

***Cell culture***

*2D culture*

As the representatives of metastatic and TNBC cell lines, MDA-MB-231 and MDA-MB-468 cell lines were cultivated in DMEM-high glucose medium (Life Technologies, Inc., Grand Island, NY, USA), which was supplemented with 10% FBS. Furthermore, the non-tumorigenic breast cell line, MCF-10A cells, were cultivated in DMEM/F12 media supplemented with 0.5 μg/ml hydrocortisone, 20 ng/ml EGF, 10 μg/ml insulin, and 5% horse serum. After sterile mammoplasty surgery, primary human fibroblast cells were extracted from a 4-mm punch made in healthy donor skin using a punch biopsy instrument. Species of skin punch were dissected into 1-mm slices and then incubated in serum-free condition media containing 100 IU/ml collagenase type I (Gibco-USA) for 30 min. Then, the cell suspension was passed through the cell strainer (pore size 100 µm). For four weeks, the isolated single cells were grown in DMEM-high glucose media with 10% FBS and 1% penicillin-streptomycin (Life Technologies, Inc.) added. After three passages, the pure fibroblast cells were obtained. Fibroblast expansion was achieved using 0.25% trypsin-EDTA for enzymatic digestion.

*Hanging drop method for spheroid (as the 3D in vitro culture model):*

A 60 mm tissue culture dish’s lid was taken off and inverted. On the inside surface of the inverted lid, droplets were placed, each containing roughly 3x103 MDA-MB-231 cells. After swiftly and carefully inverting the lid once more, it was set on top of the PBS-filled bottom chamber (serving as a hydration chamber). For four days, these culture dishes were incubated at 37 °C with 5% CO_2_ and humidity. 

***Cell uptake assay***

PKH-67 green lipid membrane dye (Sigma-Aldrich) was used to label DOX-NK-Exos following the manufacturer’s protocol. A 24-well plate was seeded with cells, and each well received 25 μg of labeled Exos. Cells were washed with PBS and fixed with 4% paraformaldehyde after a 24-hour period. After staining the nuclei with DAPI (Sigma-Aldrich), an inverted fluorescent microscope was used to examine them. On the other hand, the uptake of exosomes was quantified by flow cytometry.

***Cell viability assay***

4×10^3^ cells/100 µl were seeded in 96-well plates for 2D culture. After the cells reached 70% confluency, the wells were treated with different concentrations of DOX (0, 1, 2, 3, 4, 5 µg/ml). For 3D culture, three spheroids/100 µl in 96-well were also treated by DOX. After 48 hr, each well was incubated with methyl thiazolyl tetrazolium (MTT, 5 mg/ml in PBS; Sigma, St Louis, Missouri, USA) according to a previous study (34). Half maximum inhibitory concentration (IC_50_) was calculated for 2D and 3D cultures. Then, the treatments were conducted on 96-well plates, which were divided into NK-Exos (5 μg/well), DOX (IC_50_ concentration), DOXIL (Doxorubicin hydrochloride liposomal form, 2 mg/ml), DOX-NK-Exos (based on IC_50_ concentration), and untreated groups. MTT assay was carried out using the method described above.

***Effect of DOX-NK-Exos on the apoptosis pathway***

All groups were harvested following the protocol of the kit (Annexin-V-FLUOS Staining Kit-Roche), explained in our previous study (34). Finally, the flow cytometry method was used to identify the apoptotic population. On the other hand, all groups were harvested and mixed with Acridine Orange (AO) and Ethidium Bromide (EtBr) (100 μg/ml: 100 μg/ml). A droplet of those was placed on a slide cover with a glass coverslip. The fluorescent microscope was utilized to observe the morphological features of the cells. For the cells, the apoptosis and cell damage index were also computed.

***Gene and protein expression***

As previously described, the expression levels of p53 and VEGF-A were measured using quantitative RT-PCR (35). Briefly, total RNA was extracted using RNX^TM^-plus (Cinnagen, Iran) and complementary DNA (cDNA) synthesis was performed using Revert Aid^TM^M-MuLV RT (Fermentase). The Real-Time PCR was performed based on the Pfaffl ratio (normalized to β-actin expression level).

Cell lysate was prepared using the RIPA buffer supplemented with inhibitor cocktails of PMSF and phosphatase. The obtained cell lysate was used to measure the protein level of vascular endothelial growth factor A (VEGF-A) (34). Total protein (40 μg) was separated on the SDS-PAGE, followed by WB protocol according to the method described before (33).

***Mobility, migration, and colony formation assays***

Three experiments were carried out to evaluate how DOX-NK-Exos affected migration. For every experiment, suspended single cells were required. In 2D culture, MDA-MB-231 cells were trypsinized to produce suspended single cells, while collagenase was used to detach the spheroids in 3D culture.

For the wound healing test, the proper number of suspended single cells from 2D and 3D cultures were seeded in a 6-well plate (population doubling time (PDT) of MDA-MB-231 is 16 hr to 18 hr) to reach 70% confluency in the monolayer state. A linear wound was then scratched with a plastic pipette tip following a procedure described previously (36).

After 24 hr of starvation for the cells/spheroids, the transwell migration assay was conducted. After that, the suspended single cells from 2D and 3D were obtained and plated into the upper part of the transwell cell culture chambers (Millipore, Billerica, MA, USA). The medium containing serum was introduced as a chemoattractant into the bottom chambers. After 24 hr of incubation, the protocol was followed as described in a previous study (37). The migration data was quantified using ImageJ software.

In order to conduct the colony formation experiment, 1.5×10^3^ suspended single cells were placed in a 24-well plate. The cells were incubated for one week until colonies of considerable size were formed. Then, it was followed by the approved protocol (34).

***Statistical analysis***

For every group, every experiment was run at least three times in triplicate, and the data was processed using the SPSS program. Student’s t-test was used to establish statistical significance; an asterisk denotes significance (*P*<0.05).

## Results

***Preparation of NK cells and NK-Exos***

Twenty-one days after the seeding of NK cells, which allows the expansion of cells (Figure 1A), 87.9% of cells were single positive for CD56, 7.2% of the total population were CD56^+^CD3^+^, and nearly 3.3% were CD3^+^ only (Figure 1B). Next, the supernatant NK cells were subjected to Exos isolation using the Exo-spin^TM^ kit. TEM validated the morphology of the isolated NK-Exos, which were found as bilayer membrane structures that resembled saucers (Figure 1C). The presence of Exos-specific marker proteins (CD9 and CD63) was identified. Nevertheless, exosomes did not include the endoplasmic reticulum protein Calnexin (known as exosomal negative marker) (Figure 1D). Since Exos packaged the contents of their parental cells, the expression of specific markers on the NK-Exos were measured. In this way, CD56 expression was quantified by an anti-CD56 antibody conjugated to PE in total NK-Exos, 41.4% positive (Figure 1E). In the naïve exosomes (NK-Exos), the zeta potential was -1.8mV, and the size distribution was reported to be 131.2 nm (Figure 1F, G).

***Preparation of DOX-NK-Exos formulation***

The standard curve of DOX was drawn by different concentrations in PBS buffer as solvent (Figure 1H) (R2 = 0.99). Subsequently, other methods were employed for loading, and the highest loading efficiency of DOX was obtained as 51.29±0.36% (encapsulated/total) by ultrasonication (Figure 1I). The stability and morphology of DOX-NK-Exos were confirmed via different characterization assays. The TEM images depicted the morphology and physical characteristics (Figure 1J). A slight increase in the negative surface charge of the DOX-NK-Exos was observed (-2.0 mV in Figure 1K). The stability and integration of DOX-loaded exosomes remained unchanged, and after DOX loading, the size increased slightly to 140.5 nm (Figure 1L). DOX.HCl was entrapped inside Exos due to the hydrophilic property of the drug, which resulted in a slight increase in size. 15.4% of DOX was released from Exos at 4 hr by a dialysis membrane as the exponential release rate, while the slope of the release rate decreased up to 72 hr, which confirmed the stability and controlled release of DOX (Figure 1M). The acidic extracellular microenvironment of malignant tumors (pH = 6.5) showed higher intensity of the release, which indicates higher efficacy of the encapsulated exosomal form around the target area and tumor site. On the other hand, in the case of body serum simulation under normal physiologic conditions (pH 7.4, 50% FBS, 37 °C, and 5% CO_2_ and humidity), the release rate was 43.4% after 24 hr. These data indicated that DOX-NK-Exos were positive for exosomal markers, and DOX was efficiently loaded inside the Exos.

***DOX-NK-Exos formulation decreased the cell viability efficiently***

IC_50_ values were assessed at 1.8 µg/ml for 2D culture and slightly more for 3D culture: 2.2 µg/ml (Figure 2A), as previously reported (38). The cells/spheroids were treated with DOX-NK-Exos (in DOX concentration), DOX, and NK-Exos (5 μg/well). All exerted treatments showed cytotoxic effects after 24, 48, and 72 hr. However, no significant difference was observed between 48 hr and 72 hr for DOX-NK-Exos (Figure 2B). Therefore, 48 hr was selected for further analyses. The evaluation of DOX-NK-Exos compared to DOX and NK-Exos groups showed a significant difference (*P*<0.0*5*). Furthermore, in comparison to the untreated cells, the DAPI staining results verified the inhibitory impact of DOX-NK-Exos (Figure 2C). 

Single treatments with DOX and Exos decreased cell viability down to 88±0.9% and 49±1.7%. However, the combined formula resulted in a more severe reduction of cell viability (down to 31±0.6%) due to its possible additive effect. Indeed, the exosomal encapsulation containing 2.2 µg/ml DOX was more efficient than liposomal formulations (at the same concentration of the effective material) in the 3D mimicking environment (Figure 2D).

Treatment of noncancerous cell lines demonstrated the lowest cytotoxicity against noncancerous normal cell lines and preferential cytotoxicity against cancer cells. Treatment of the fibroblasts and MCF-10A cells with free DOX showed the highest cytotoxic effect. In contrast, only 12.7% and 18.8% decrease in cell viability were observed when fibroblasts and MCF-10A were treated with DOX-NK-Exos, respectively. These results indicated that DOX-NK-Exos selectively reduced the viability of tumor cells and spared normal cells. Hence, DOX-NK-Exos could be more effective in treating cancer by reducing nonspecific toxicity than free DOX. In all treatment groups, fibroblast cells showed higher viability than MCF-10A cells (Figure 2E). The statistical analysis of DOX and NK-Exos groups in comparison to DOX-NK-Exos showed a significant difference (*P*=0.007 and 0.01, respectively).

***In vitro uptake of DOX-NK-Exos by fluorescent labeling***

After treatment with DOX-NK-Exos, fibroblast (Figure 3A) and MCF-10A (Figure 3B) cells were observed with a fluorescence microscope. In the same way, DOX and DOX-NK-Exos treated MDA-MB-231 in 2D culture (Figure 3C, D) and 3D spheroids (Figure 3E, F) were also observed. The red fluorescent intensity due to the presence of DOX in the cells treated with DOX-NK-Exos was higher than that of DOX alone, indicating that DOX-NK-Exos can be more effectively taken up by the cells in both 2D and 3D cultures (Figure 3D, F). Figure 4G simultaneously showed the presence of DOX in the nuclei and the uptake of Exos into the cell membranes, followed by the binding of PKH-67-labeled DOX-NK-Exos to the cells. 

The flow cytometry analysis of DOX and DOX-NK-Exos treated groups revealed 65.4% and 73.3% positivity in the PI channel (red based on DOX presence), respectively. The binding of NK-Exos to cells was measured as a negative control (0.16%). Compared to the free drug, Exos-containing cargo showed a significantly higher rate of uptake (Figure 3H).

***DOX-NK-Exos affected cell apoptosis***

DOX-NK-Exos treatment group revealed remarkable induction of apoptosis. On the other hand, NK-Exos induced the apoptosis pathway due to the properties of parental cells in the induction of apoptosis (Figure 4A). The graph shows the total early and late apoptosis in DOX-NK-Exos group compared to both NK-Exos and DOX (Figure 4B). 

In dual AO /EtBr staining, the fluorescent color for viable cells with green chromatin and organized structures was predominantly green. They have normal nuclei staining, but early to late apoptotic (even dead) cells showed orange to red fluorescence. As shown in Figure 4C, DOX (in both free DOX and DOX-NK-Exos treated groups) killed the cells, which was indicated by the red color under the bright-field microscope and also the blue color by trypan blue (a viability staining). The morphology of the NK-Exos-treated cells changed markedly, and the cells moved toward apoptosis. The live cells in the untreated groups showed strong green fluorescence, and none was blue under the microscope. 

The number of living cells glowing green was counted in 10 high-power fields, and the mean percentage of living cells was reported (Figure 4D). The proportion of live cells in the DOX-NK-Exos treated group was significantly lower than the live cells in the free DOX-treated group (*P*<0.0*5*).

As shown in Figure 4E, DOX-NK-Exos was effectively taken up by the MDA-MB-231 spheroids and led to cell death in the outer layer, which eventually expanded to the inside of the spheroids. The morphology of the spheroids was markedly altered in both DOX and DOX-NK-Exos treatments. They were no longer transparent like an untreated group, and the spheroidal mass cells detached from the central mass.

***DOX-NK-Exos suppressed the mobility and migration***

Figures 5A and B showed that scratch-wound healing was slower in the DOX-NK-Exos treated groups. The treatment with both DOX and NK-Exos (DOX-NK-Exos) led to a significant increase in wound width and a decrease in cell proliferation compared to the untreated cells. The transwell migration assay also confirmed this finding. As illustrated in Figures 5C and D, the same effects were observed. In comparison to the solitary treatments of Exos or DOX, the residual limited population had much less capacity for mobility/migration following the DOX-NK-Exos therapy. This could be attributed to the simultaneous presence of NK-Exos and anti-invasion drugs/chemotherapeutics such as DOX, which suppressed the migration of MDA-MB-231 in the transwell assay. These provocative findings revealed that the mobility and migration of cells obtained from 3D spheroids was greater than that of cells derived from 2D settings, indicating the existence of aggressive and resistant cells in the center of tumor-like spheroids that may represent cancer stem cells (33) (Figure 5B, D).

***DOX-NK-Exos suppressed the colony formation of the breast cancer cells***

DOX-NK-Exos treatment also had a more long-term effect on the cells compared to the NK-Exos (Figure 6A, C). This treatment significantly suppressed the colony formation of breast cancer cells and reduced cell proliferation. The colonies of the untreated group were very dense compared to the DOX-NK-Exos treated group; even the cell boundaries were not easily discernible, and the condition of the clones resembled a three-dimensional solid tumor (Figure 6B).

***Gene and protein expressions***

The results obtained from the Real-Time PCR (Figure 6D) revealed that VEGF-A gene expression was down-regulated in the groups treated with DOX and DOX-NK-Exos. While the VEGF-A expression was slightly reduced in the NK-Exos treated group (*P*<0.0*5*). The protein level of VEGF-A revealed the ability of DOX-NK-Exos to reduce the expression of this protein (Figure 6E). One of the most common genetic abnormalities in TNBC is the loss or mutation of the p53 tumor suppressor gene (39, 40). The p53 gene expression was up-regulated in all treated groups, but treatment of cells with DOX-NK-Exo led to more increased expression of p53.

## Discussion

Hitherto, splendid advances have been made in encapsulating chemotherapeutic agents within nanoscale carriers (10–100 nm in diameter) for cancer treatment. However, effective drug accumulation in the tumor site without toxicity and immune activation remains an ongoing challenge (10). Tissue-specific, safe, and non-immunogenic delivery technologies are essential for the clinical implementation of these systems. Exosomes have been widely used for targeted delivery of various chemotherapeutic agents to specific tissues and cells. Han *et al*. have entrapped hydrophobic PTX using NK-Exos to enhance its antitumor effect and significantly inhibited the proliferation of breast cancer cells (41). The main components of NK-Exos are cytotoxic proteins, cytokines, and microRNAs (42). Apart from their anticancer properties, immune cells are reported to have immunomodulatory effects upon stimulation. Numerous investigations have documented the role of NK-Exos in reversing immunosuppression, indicating their possible application in cancer immunotherapy. (42).

Exosomes of NKs are isolated from the supernatants of NK92, *ex vivo* cultured NK cells (41, 43), resting NK cells (29), NK cell-enriched lymphocytes (44), and human plasma (45). The surface markers of exosomes derived from resting NK cells are the same as those of activated NK cells (29). Activated NK cells are an exciting source of exosomes due to their ability to release large exosomes. Therefore, we have isolated and characterized our exosomes from NK cells to exploit their potency for cargo delivery. Characterization of NK-Exos indicated that NK-Exos were 100–150 nm in size, had a core-shell structure, and displayed CD63 and CD9 as putative exosome markers. The absence of the Calnexin marker on the purified exosomes indicated that they were not derived from the endoplasmic reticulum pathway. The expression of the CD56 marker reaffirmed that they are isolated from NK cells. 

DOX was loaded into the inner compartment of exosomes using Freeze/thaw, incubation, and ultrasonication methods. With the last method, we achieved an acceptable efficiency, which was comparable to those published by others. Tian *et al*. used mouse exosomes derived from immature dendritic cells (imDCs) to deliver DOX to tumor tissue. DOX was loaded into the exosomes by electroporation, and the encapsulation efficiency was reported to be 20%. Gomari *et al*. have achieved low encapsulation efficiency in MSC-derived exosomes (17). In this regard, we arrived at an encapsulation efficiency of about 50% using the ultrasonication method. This yield is consistent with the results of the study conducted by Kim *et al*. They have found that the highest incorporation efficiency (~30%) of PTX into exosomes was obtained by mild sonication compared with electroporation and incubation (46). Electroporation is a harsh loading method that could compromise the integrity of exosome membranes, while we observed that the ultrasonication method did not significantly alter the integrity of exosomes. Therefore, their ability to interact and enter the cells remained intact. 

DOX-NK-Exos had no significant changes in morphology and size. In line with prior studies, accommodation of loaded drug slightly increased the Exos size, and no considerable aggregation (12). Moreover, the surface charge of DOX-loaded exosomes was diminished compared to the naïve exosomes (-2.0 vs -1.8). Similar observations have been reported (47), which can partly be explained by the rearrangements of the exosome’s phospholipid bilayers upon ultrasonication. 

Drug release and stability confirmed the slow and controlled release of DOX, structural stability of the exosomes, and successful DOX loading during ultrasound treatment. These observations are consistent with a previous study, in which DOX was loaded with ultrasonication, and 3% of DOX was retained even at the end of 72 hr incubation (47). Some studies have reported that the release of DOX-loaded exosomes is pH-dependent (14, 48). Researchers have revealed that ultrasonication would accumulate the acidic hydrophilic drugs (such as DOX hydrochloride (DOX.HCl), which we used here) in the inner acidic space of exosomes. They have shown that the Acridine Orange (AO) loaded exosomes were more efficiently taken up by the target cells than the free AO (49). 

The DOX-NK-Exos formulation as a drug delivery system (DDS) demonstrated a greater inhibition rate in MDA-MB-231 human breast cancer cells than free DOX at the same dose, based on the antitumor studies. This result is consistent with other antitumor studies, which have used the NK-Exos potency for drug delivery (41). It could be deduced that the infiltration of free DOX within cells is less than the DOX released by NK exosomes. In agreement with some other studies (17), the higher cellular toxicity of DOX delivered by exosomes might be due to the controlled release and structural stability of the encapsulated DOX. Exosomes’ lipid bilayer has been shown to be able to directly target and fuse with cell membranes, improving the drug’s cellular internalization and boosting its therapeutic efficacy. This concept is supported by several studies indicating that DOX-Exos significantly decreased the tumor growth rate in a mouse model of breast cancer (17, 50).

MDA-MB-231 cells can form a spherical structure via the interactions of collagen I/integrin β without cadherin involvement (51). Consistent with the other experiments, the cell viability assay for spheroids showed that DOX-NK-Exos accounts for the highest toxicity against MDA-MB-231 cells compared to NK-Exos and free DOX for 72 hr. Our results suggested controlled and slow release of exosomes with no changes in toxicity for 72 hr. Our experiments also showed that DOX-NK-Exos could maintain high intracellular concentrations over an extended incubation. Furthermore, compared to free DOX at the same dose, the inhibitory effect of DOX-NK-Exos on the growth of MDA-MB-231 cells was more prominent. Receptor-mediated endocytosis, phagocytosis, micropinocytosis, or direct fusion with the plasma membrane of MDA-MB-231 spheroids are effective ways to uptake DOX-NK-Exos. They can lead to cell death in the outer layer of the spheroids and eventually their inside. These findings suggest that cancer treatment via Exos loaded with cytotoxic drugs was more feasible and profitable. These observations corroborated the previously reported results by Han *et al*. They have used the NK-Exos to deliver PTX into the MCF-7 cells (41). 

The apoptotic results demonstrated the greater cytotoxicity of DOX-NK-Exos in comparison to free DOX, which was consistent with the cell viability assay. The increased cellular toxicity of DOX-Exos is probably due to the higher intracellular DOX levels that these molecules acquire. (49). The distribution pattern by fluorescence microscopy revealed that DOX-NK-Exos were rapidly distributed in the cytoplasm. Proteoglycans, integrins, and lectins are examples of adhesion proteins that aided in the initial attachment to recipient cells and probably aided in the rapid uptake of cells (52). Researchers have reported the diffused pattern of free DOX in the cytoplasm and nucleolus compared to the discrete spots of the liposomal DOX and DOX-Exos (12). In line with our flow cytometry results, they demonstrated that DOX-Exos uptake was more efficient with high accumulation in endocytic structures. This observation is corroborated by other studies, which showed that DOX-Exos had induced a higher rate of apoptosis compared to free DOX following cellular uptake. More interestingly, they have reported that the uptake of exosomes is much faster and more efficient than liposomal formulations. Our findings further support the notion that DOX-Exos can preserve the high intracellular drug levels over a considerably prolonged incubation time (72 hr). According to earlier research, unlike free DOX, DOX-Exos is not susceptible to efflux through multidrug resistance (MDR) transporters. This fact would explain the increased cellular levels after 4 hr of incubation (46). 

Down-regulation of the VEGF-A gene in the presence of NK-Exos suggested that Exos could enhance the cellular uptake and targeting effects of DOX to the breast cancer cells and improve the efficiency of its anti-angiogenesis functions. This protein creates a pathway for invasion because it causes the development of tumor-associated blood vessels (53). Consistent with other studies, the expression level of the pro-apoptotic p53 gene was significantly increased after exposure to various doses of DOX (54, 55). The MDA-MB-231 cell line divides rapidly and has an aberrant p53 signaling pathway. These properties most likely enhance DOX’s toxic effect on DNA replication (56). 

One of the significant clinical drawbacks of DOX is its nonspecific toxicity against cardiomyocytes, which could lead to acute cardiotoxicity even at very low doses. Our results revealed that despite higher efficacy in killing tumor cells, DOX-NK-Exos have lower toxicity against normal fibroblast and MCF-10A cells. When non-cancer cells were treated, DOX-NK-Exos was able to spare normal cells while specifically reducing the viability of tumor cells. Similar results were obtained by Srivastava *et al*. (57) and Toffoli *et al.* (58). They showed that exosome-mediated DOX had better biodistribution and efficacy and lower toxicity to normal tissues. Toffoli *et al*. (58) have also observed low cardiotoxicity for DOX-Exos in a mice study. Thus, our outcomes strongly recommended a method to bypass cardiotoxicity while enabling tumor killing. Although the study results are promising, they should be considered cautiously due to the unknown *in vivo* cardiotoxicity of NK-derived exosomes. Studies are currently underway in our laboratory to test the efficacy of NK-derived exosomes *in vivo* in a breast cancer xenograft model.

## Conclusion

It could be concluded that combining the natural tropism of exosomes toward tumors (due to the enhanced permeability, EPR, and retention effect) and the natural features of NK cells, DOX-NK-Exos could be a highly favorable cytotoxic bionanomaterial agent against 2D and 3D cultures of MDA-MB-231 cancer cells. Moreover, DOX-NK-Exos could readily be engineered to target specific markers. On the other hand, this treatment could significantly reduce the ability of tumor-derived cells to form a new colony or abnormal mass. To the best of our knowledge, this is the first report to demonstrate the antitumor effects of DOX-NK-Exos on human breast cancer mass (3D culture) as the tumor mimic. The residual cells derived from spheroids indicated the presence of a self-renewing property and were more aggressive, similar to the cancer stem cells within the tumor. However, further *in vivo* studies are required to determine if the anticancer action of NK-Exos or the elevated cellular amounts of DOX is responsible for this inhibitory impact.

It should also be noted that exosomes play a vital role in facilitating cell-to-cell (whether normal, malignant, or cancerous cells) communication by transporting various payloads, including proteins, RNAs, and DNA, from one cell to another. Therefore, exosomes from different sources can influence cancer conditions either positively or negatively and can contribute to phenomena such as cancer progression, metastasis, immune responses, and therapy resistance. Thus, exosomes could be responsible for significant therapeutic implications. In particular, exosomes derived from mesenchymal stem cells can lead to immunomodulatory effects, which could be beneficial for treating autoimmune diseases. However, it could bear adverse effects on the immune system fighter’s standby mode in incurable diseases, especially cancer. Given these circumstances, depending on the purpose, the type of exosome source should be different. While exosome therapies hold significant promise, they are also confronted with potential limitations, such as inadequate zeta potential, limited half-life, and the absence of standardized procedures for exosome isolation and purification and also biopharmaceutical regulation. Despite these limitations, exosomes could be employed as biomarkers and drug delivery systems, and exosome-loaded scaffolds can be used for regenerative medicine.

## Data Availability

The data generated during the study to support the findings are available upon request from the corresponding author (Dr. Zahra Sadat Hashemi and Dr. Ramin Sarrami Forooshani) upon reasonable request.
